# Brain age predicted using graph convolutional neural network explains neurodevelopmental trajectory in preterm neonates

**DOI:** 10.1007/s00330-023-10414-8

**Published:** 2023-11-14

**Authors:** Mengting Liu, Minhua Lu, Sharon Y. Kim, Hyun Ju Lee, Ben A. Duffy, Shiyu Yuan, Yaqiong Chai, James H. Cole, Xiaotong Wu, Arthur W. Toga, Neda Jahanshad, Dawn Gano, Anthony James Barkovich, Duan Xu, Hosung Kim

**Affiliations:** 1 School of Biomedical Engineering, Sun Yat-Sen University, Shenzhen, 518107 China; 2 Department of Neurology, USC Stevens Neuroimaging and Informatics Institute, Keck School of Medicine, University of Southern California, 2025 Zonal Ave, Los Angeles, CA 90033 USA; 3 Guangdong Key Laboratory for Biomedical Measurements and Ultrasound Imaging, School of Biomedical Engineering, Medical School, Shenzhen University, Shenzhen, 518060 China; 4 Division of Neonatology, Department of Pediatrics, Hanyang University, Seoul, Korea; 5 Centre for Medical Image Computing, Department of Computer Science, University College London, London, UK; 6 Departments of Neurology and Pediatrics, University of California, San Francisco, San Francisco, CA USA; 7 Department of Radiology & Biomedical Imaging, University of California, San Francisco, San Francisco, CA USA

**Keywords:** Brain age prediction, Graph convolutional network, Preterm neonates, Brain morphology, Structural equation modelling

## Abstract

**Objectives:**

Dramatic brain morphological changes occur throughout the third trimester of gestation. In this study, we investigated whether the predicted brain age (PBA) derived from graph convolutional network (GCN) that accounts for cortical morphometrics in third trimester is associated with postnatal abnormalities and neurodevelopmental outcome.

**Methods:**

In total, 577 T1 MRI scans of preterm neonates from two different datasets were analyzed; the NEOCIVET pipeline generated cortical surfaces and morphological features, which were then fed to the GCN to predict brain age. The brain age index (BAI; PBA minus chronological age) was used to determine the relationships among preterm birth (i.e., birthweight and birth age), perinatal brain injuries, postnatal events/clinical conditions, BAI at postnatal scan, and neurodevelopmental scores at 30 months.

**Results:**

Brain morphology and GCN-based age prediction of preterm neonates without brain lesions (mean absolute error [MAE]: 0.96 weeks) outperformed conventional machine learning methods using no topological information. Structural equation models (SEM) showed that BAI mediated the influence of preterm birth and postnatal clinical factors, but not perinatal brain injuries, on neurodevelopmental outcome at 30 months of age.

**Conclusions:**

Brain morphology may be clinically meaningful in measuring brain age, as it relates to postnatal factors, and predicting neurodevelopmental outcome.

**Clinical relevance statement:**

Understanding the neurodevelopmental trajectory of preterm neonates through the prediction of brain age using a graph convolutional neural network may allow for earlier detection of potential developmental abnormalities and improved interventions, consequently enhancing the prognosis and quality of life in this vulnerable population.

**Key Points:**

*•Brain age in preterm neonates predicted using a graph convolutional network with brain morphological changes mediates the pre-scan risk factors and post-scan neurodevelopmental outcomes.*

*•Predicted brain age oriented from conventional deep learning approaches, which indicates the neurodevelopmental status in neonates, shows a lack of sensitivity to perinatal risk factors and predicting neurodevelopmental outcomes.*

*•The new brain age index based on brain morphology and graph convolutional network enhances the accuracy and clinical interpretation of predicted brain age for neonates.*

**Supplementary information:**

The online version contains supplementary material available at 10.1007/s00330-023-10414-8.

## Introduction

Predicted brain age (PBA) derived from neuroimaging and machine learning approaches has emerged as a biologically meaningful index that indicates the status of brain development or aging [1]. Estimation of neonatal brain age using the PBA measurement may be clinically useful in evaluating neurodevelopment and the effects of perinatal factors. Studies have demonstrated that brain structural and functional maturation is impaired by preterm birth, resulting in decreased cerebral volume [2], altered cortical surface area [3] and microstructural organization [4], and aberrant functional and structural connectivity [5, 6]. Moreover, various perinatal factors, such as birthweight, brain injuries, gestational age, and chronic lung disease of prematurity (CLD), also termed bronchopulmonary dysplasia, affect brain development and neurodevelopmental outcomes [7]. Thus, it remains clinically imperative to identify robust metrics that assess how various perinatal factors affect neurodevelopmental outcomes. PBA, with its increasing recognition in biological relevance [8, 9], may provide such clinical utility in assessing preterm brain development throughout the third trimester.

To derive the PBA measurement, studies to date have employed machine learning and deep learning (DL) methods [10, 11] that reveal important features without prior information or hypotheses. For instance, in aging brains, the PBA has been investigated using standard DL approaches [12] where an image volume is inputted into a convolutional neural network (CNN) and a number representing the whole brain age is outputted [13, 14]. However, these PBA models that were applied to structural connectivity data [15, 16] and myelin-based brain features [17] showed a lack of sensitivity in predicting neurodevelopmental outcomes [18].

Exploring specific morphological features could potentially boost the clinical significance of PBA, given that the third trimester is characterized by morphological changes that are highly sensitive to both age and pathology [19, 20]. Therefore, in contrast to previous brain age prediction strategies that directly applied DL models on MR images, we specifically focused on highly age- and pathology-related features during the third trimester, such as increases in cortical folding or volume, to enhance the clinical interpretation of neonatal brain age prediction. Additionally, numerous edges that connect neighboring vertices (i.e., points on the cortical surface) contain valuable topological information [21], which represents the location and adjacency among neighboring vertices. In this regard, a Graph Convolutional Network (GCN) model could be more suitable for the analysis of cortical morphological features, as it incorporates these topologies.

A brain age index (BAI; PBA minus chronological age) in neonates may indicate accelerated or delayed neurodevelopment. *We hypothesize that the BAI at neonatal scan reflects the collective effects of pre-scan (pre- and postnatal) clinical factors*, including preterm birth, perinatal brain injuries, postnatal treatments, neonatal infections, and postnatal cardiorespiratory complications. *We also hypothesize that the BAI can predict neurodevelopmental outcomes*. Furthermore, *we hypothesize that these relationships with the BAI can only be uncovered when morphological features are effectively incorporated into the prediction model.* Considering these, we explored the accuracy and clinical utility of PBA using a GCN model [22]. Cortical thickness, sulcal depth, and GM/WM intensity ratio [23] maps were extracted from the cortical mesh and inputted to the GCN. To evaluate the GCN-based PBA, we assessed (1) whether the PBA using GCN reflects the influence of pre-scan clinical factors on neurodevelopment; (2) whether BAI of neonatal MRI is a sensitive predictor of neurodevelopmental outcome at 30 months; (3) whether BAI at scan mediates the relationship between preterm birth-related clinical factors and neurodevelopmental outcome at 30 months; (4) whether the GCN approach outperforms other deep-learning brain age prediction algorithms that can clinically interpreting the developmental trajectories of preterm neonates.

## Methods

### Subjects

Our dataset comprised 129 preterm neonates (gestational age [GA] at birth range 24–33 weeks) admitted to UCSF (University of California at San Francisco) Benioff Children’s Hospital between June 2008 and May 2017 (Table 1) and 407 neonates from the developing Human Connectome Project (dHCP; http://www.developingconnectome.org/; GA at birth range 24–42 weeks; Table 2). Most subjects from UCSF were scanned twice, but some scans were excluded due to a large amount of motion artifact, resulting in a total of 170 MRI scans (mean postmenstrual age [PMA] range at 1^st^ scan: 26.7–35.7 weeks; 2^nd^ scan: 32.1–43.4 weeks). Parental consent was obtained through a protocol approved by the Institutional Committee on Human Research. In the dHCP cohort, the MRI images acquired from only singletons were included in this study. The images were visually inspected, and images with substantial motion on MRI or major focal parenchymal lesions at the time of their scan were excluded. The final dHCP sample consisted of 407 (187 female) neonates with a PMA of 29 to 45 weeks (mean PMA: 39.7 ± 3.1 weeks) in age at the time of the scan.

**Table 1:** Demographic and clinical characteristics for preterm neonates admitted to UCSF

Demographic	
Subjects (*n*)	129
MRI scans (*n*)	170
Sex: male (*n*)	70
GA at birth (weeks, mean ± SD)	28.2 ± 1.9
Weight at birth (gram, mean ± SD)	1080 ± 311.2
PMA at MRI	
1^st^ scans (*n* = 129)	31.4 ± 1.9
2^nd^ scans (*n* = 41)	36.0 ± 1.9
Characteristic^a^	Number (%)
*Maternal/antenatal factors*	
Maternal age, yrs	29.8 ± 6.5
Placenta previa	11 (8.5)
Drug abuse^b^	11 (8.5)
Magnesium sulfate	80 (62.0)
Exposure to prenatal steroids	109 (84.5)
Chorioamnionitis	13 (14.0)
*Delivery / Perinatal factors*	
Twin	58 (44.9)
Caesarean section delivery	78 (60.5)
* Postnatal factors*	
Exposure to postnatal steroids	13 (14.0)
Hypotension	76 (58.9)
Infant infection	70 (54.2)
Patent ductus arteriosus	65 (50.4)
Necrotizing enterocolitis^c^	5 (3.9)
Duration of intubation, days	9.1 ± 13.6
Chronic lung disease	36 (27.9)
* Neurodevelopmental outcome (Bayley scales III) in 30 months*^d^	
Cognitive score	103.0 ± 15.45
Language score	92.6 ± 14.0
Motor score	93.7 ± 13.2

^a^ Data presented as number (%), or mean ± standard deviation. ^b^ All subjects with maternal smoking (based on self-report) were exposed to marijuana, two were also exposed to tobacco. ^c^ It was diagnosed based on Bell’s stage II criteria [24]; ^d^ Scores were derived from 38 preterm subjects revisiting the center at 30 months after their birth. PMA: postmenstrual age

**Table 2:** Demographic characteristics for neonates admitted to dHCP dataset

Demographic	
Subjects (*n*)	407
MRI scans (*n*)	407
Sex: male (*n*)	220
GA at birth (weeks, mean ± SD)	38.2 ± 3.9
PMA at MRI	39.7 ± 3.0
Weight at birth (gram, mean ± SD)	1080 ± 804.2

### MRI acquisition and image processing

Newborns enrolled until 2011 (*n* = 56) were scanned on a 1.5-Tesla General Electric Signa HDxt system using a specialized high-sensitivity neonatal head coil built within a custom-built MRI-compatible incubator. T1-weighted images were acquired using sagittal 3-dimensional inversion recovery spoiled gradient echo (3D SPGR) (TR = 35; TE = 6; FOV = 256 × 192 mm^2^; number of excitations [NEX] = 1; and FA = 35°), yielding images with 1 × 1 × 1 mm^3^ resolution. Newborns enrolled between 2011 and 2017 (*n* = 73) were scanned on a 3-Tesla General Electric Discovery MR750 system. T1-weighted images were acquired using sagittal 3D IR-SPGR (inversion time = 450 ms; FOV = 180 × 180 mm^2^; NEX = 1; FA = 15°), yielding images with 0.7 × 0.7 × 1 mm^3^ resolution.

All T1 images from dHCP dataset were acquired on a Philips Achieva 3.0-T scanner using a 32-channel neonatal head coil [25]. All images were collected using an IR (inversion recovery) TSE sequence with the same resolution with TR = 4.8 s, TE = 8.7 ms, SENSE factor 2.26 (axial) and 2.66 (sagittal). Motion correction and super-resolution reconstruction techniques were employed, resulting in isotropic volumes of resolution $0.5\times 0.5\times 0.5 \;{\mathrm{mm}}^{3}$. All images were collected as part of the dHCP and are described in detail in Makropoulos et al [26].

Cortical surfaces were constructed using NEOCIVET-v3 [27–29] (Methods S2). Key steps in the pipeline include image denoising, intensity nonuniformity correction, skull-striping, tissue segmentation, surface reconstruction, and surface registration. All major steps are specifically designed for neonatal MRI. Cortical morphology was quantitatively characterized by measuring cortical thickness, sulcal depth, and GM/WM intensity ratio [23] on the cortical surface at 81,924 vertices (163,840 polygons). Cortical features were harmonized using the ComBat method [30] prior to GCN training. A comparison of cortical features before and after the harmonization can be found in Figure S1. The validation of segmentation and surface construction quality using NEOCIVET is described in Figure S2.

### Clinical factors and neurodevelopmental assessment

Neonatal demographic and clinical variables are described in Tables 1 and 2. Clinical variables are not publicly available for dHCP, so clinical analyses were only conducted on the UCSF dataset. Newborns with culture-positive sepsis, clinical signs of sepsis with negative blood culture, or meningitis were classified as having an infection. Newborns with clinical signs of patent ductus arteriosus (PDA; prolonged systolic murmur, bounding pulses, and hyperdynamic precordium) and evidence of left-to-right flow through the PDA on echocardiogram were classified as having a PDA. Necrotizing enterocolitis (NEC) was diagnosed according to Bell stage II criteria or higher.

To assess neonatal brain injuries, two pediatric neuroradiologists (A.J.B., H.J.L.) blinded to patient history reviewed individual MRI scans from UCSF and dHCP, including 3-D T1 and axial T2-weighted sequences. Severity of three leading drivers of neurodevelopmental deficits, i.e., intraventricular hemorrhage (IVH), ventriculomegaly (VM), and periventricular leukomalacia (PVL) or white matter injury, were visually scored (Methods S1). Details on brain injuries are listed in Tables S1-2. In the current study, we merged infants with mild injuries and those with no injury into one none-mild injury group, since the two groups exhibited no significant differences in the following analyses.

All the infants from UCSF were referred to the UCSF Intensive Care Nursery Follow-Up Program upon discharge for routine neurodevelopmental follow-up. Neurodevelopment was assessed using the Bayley-III, which was performed by unblinded clinicians at 30 months’ age corrected to 40 weeks, to assess cognitive, verbal/language and neuromotor performance. Follow-up was available in 38 of the 129 infants who survived to hospital discharge.

### Brain age prediction

**Fig. 1:**
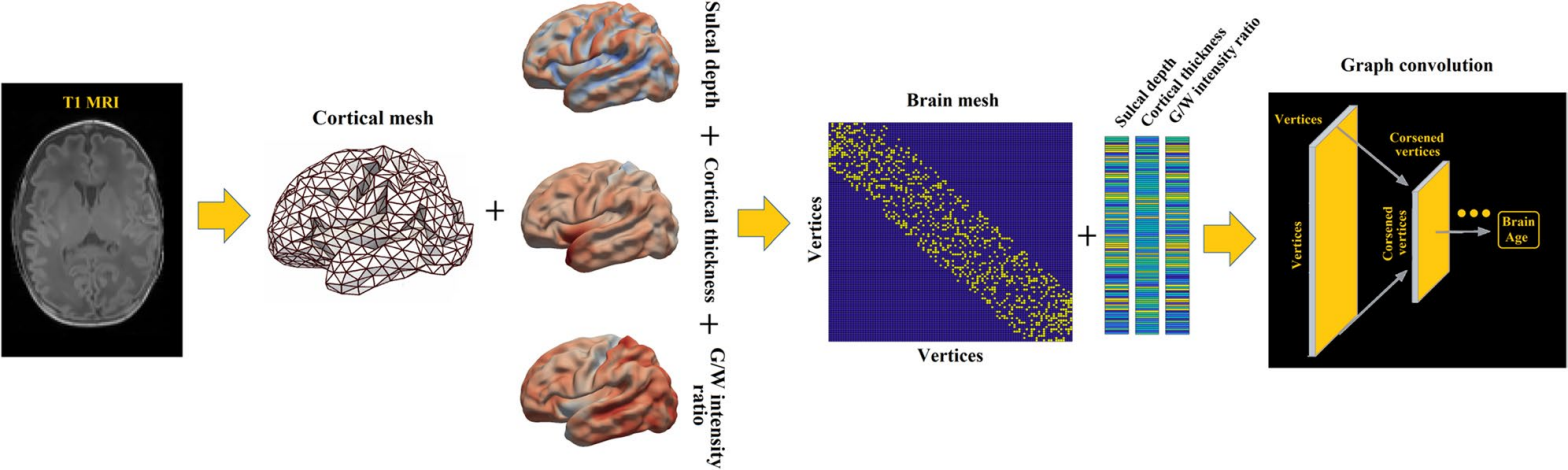
The proposed graph-based convolutional network for brain age prediction. Importantly, we did not use topology-varying surfaces because of the nature of the GCN model applied in this study. Rather, we considered cortical morphological changes that occur in relation to brain size and gyrification using cortical thickness, sulcal depth, and GM/WM intensity ratio. The GCN employed in our study requires identical graph/mesh structures for all individual inputs, while the features on nodes/vertices can vary

The proposed PBA model using GCN is illustrated in Fig. 1. GCNs [22] are designed to exploit the underlying graph structure of the data (Methods S3). We down-sampled 81,924 vertices on cortical surfaces to 1284 vertices using the icosahedron downsampling to investigate the prediction accuracy while saving computational time in training the GCN (Methods S4 and Figure S3). The input graphs combined the harmonized cortical features as the nodal features at 1284 vertices, with a sparse binary adjacency matrix representing the mesh topology, by which the edge in the graph was defined as the connections between each vertex and its neighbor vertices. GCNs consider spectral convolutions on graphs defined as the multiplication of a signal by a filter in the Fourier domain, which is approximated by Chebyshev polynomials. In the pooling operation, the vertices of the graph are rearranged to form a 1D pooling to ensure efficiency. The GCN used in this study consists of three convolutional layers and three pooling layers.

To compare our cortical surface-based GCN model with the conventional image-based DL model, we also built a PBA model by applying a CNN model [31] to T1 MR images directly (Methods S5). This model was fed a 3D scan as input and encoded each image slice using a 2D-CNN encoder. Next, it combines the slice encodings using an aggregation module, resulting in a single embedding for the scan. This model has been proven to be better than the conventional 3D-CNN-based model in adult brain age prediction study [31].

We split the data into *k* = 5 groups (folds), with 20% of data used for testing at each fold. The remaining 80% of the data were further iteratively split for training (64%) to fit the models and validation (16%) to tune the hyperparameters (in Methods S7 and Figure S4).

We added a bias correction as previously described in [12, 32] to correct age dependency of the training residuals. Briefly, we used a linear model $PBA=\alpha *{PBA}^{\prime}+\beta$ to obtain an unbiased estimate of $PBA$ as ${PBA}^{\prime}=(PBA-\beta )/\alpha$, where the parameters α and βare estimated during training (on both the combination of training and validation set) and are thus applied directly to the test set. After calculating PBA for each subject, we further calculated a metric, brain age index (BAI), that reflected a subject’s relative brain health status. BAI was measured by subtracting the postmenstrual age at the time of the MR scan from the unbiased ${\mathrm{PBA}}^{\prime}$ [14].

### Statistical analysis

To validate our hypothesis that perinatal clinical factors negatively affect brain growth (lower BAI), each variable was dichotomized using clinically defined categorization or median if arbitrary (Table S1). Necrotizing enterocolitis was not included due to the small sample size (*n* = 5). We then tested the group difference in BAI for each variable separately in a univariate fashion while correcting for PMA at scan and other clinical factors, using a general linear mixed-effect model that addressed changes of within- and between-subject effects and removed the effects from covariates other than the main variable.

**Fig. 2:**
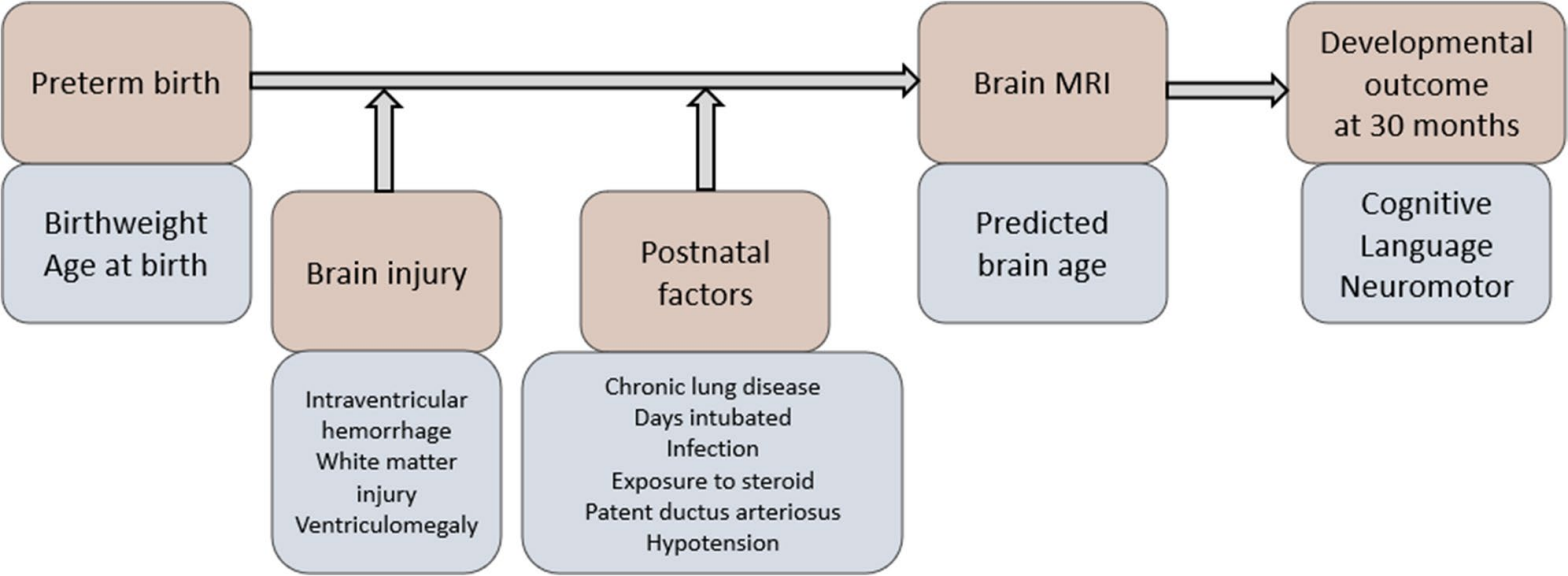
Hypothesized clinical risk factors and timeline after birth in relation to brain development in preterm infants. Orange: different time stages of the conditions, procedures, or measures; Blue: characteristics of the conditions, procedures, or measures

Moreover, we built structural equation models (SEM) that impute relationships between latent variables (Methods S9). Based on the hypothesized latent risk variables and timeline in Fig. 2, we analyzed multiple relationships/paths between severity of preterm birth, perinatal injuries, pre-scan postnatal factors, BAI at postnatal scan, and neurodevelopmental outcome scores at 30 months. This analysis, which was designed to identify the clinical variables and their paths leading to adverse neurodevelopmental outcomes, was conducted only on 50 MRI images from the UCSF dataset, including baseline and follow-up scans at 30 months from 38 preterm survivors.

We executed our GCN and CNN scripts with Pytorch 1.14.0. The SEM was executed using IBM SPSS Amos v24. Brain age bias correction and other statistics were performed using MATLAB 2021a. The brain age prediction code is available at https://github.com/bigting84/Brain-Age-Prediction. Anonymized data is available at https://github.com/bigting84/Neonatal-surface.

## Results

### Performance of GCN-based age prediction

**Fig. 3:**
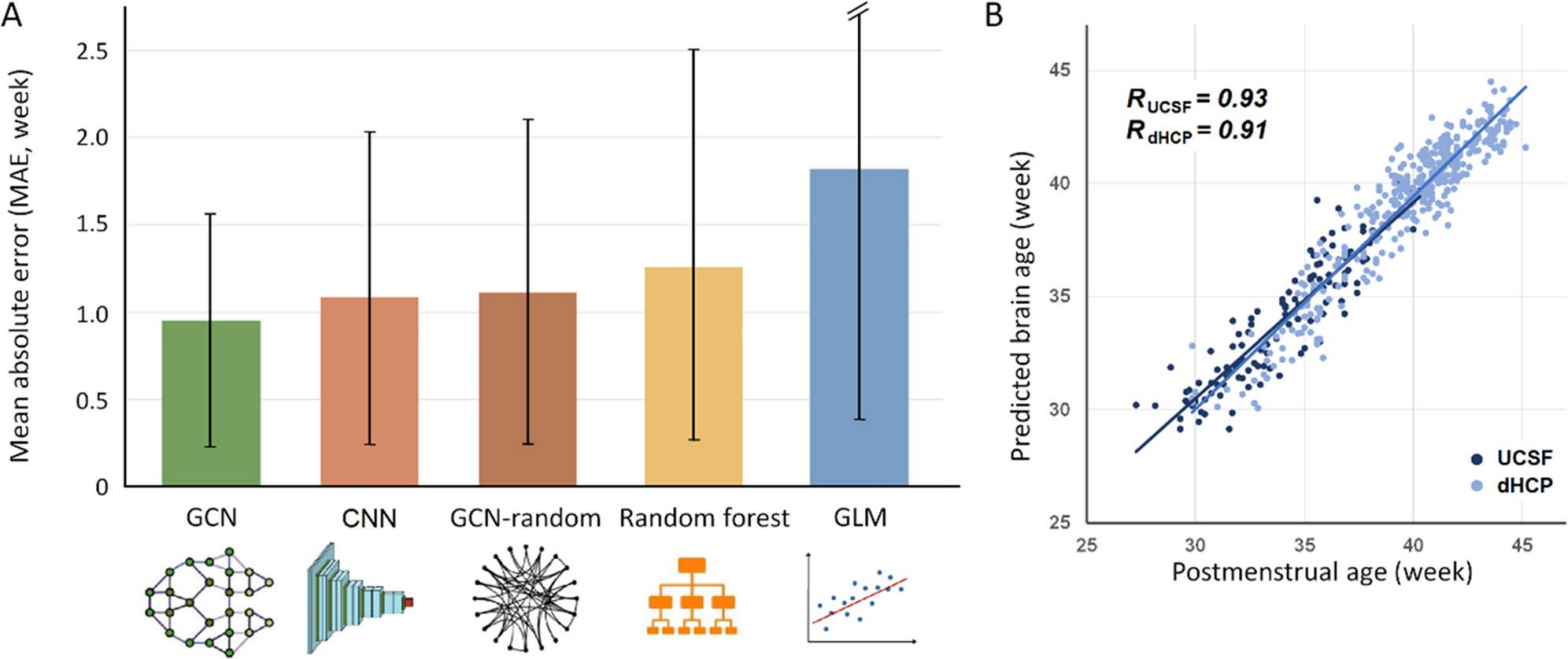
**A** Comparison of brain age prediction errors among regression models. The height of each bar indicates the mean MAE, and the black line indicates the standard deviation of MAE per model. GCN demonstrates the best prediction results. **B** Scatter plot displaying PBA using GCN model *vs.* chronological brain age. We achieved a correlation coefficient r of 0.94 using GCN, which was significantly higher than the errors of the other models (GCN: MAE = 0.963 weeks, *r* = 0.94; CNN: MAE = 1.05 weeks, *r* = 0.89; GCN on the random connections: MAE = 1.1 weeks, *r* = 0.89; RF: MAE = 1.27 weeks, *r* = 0.85; GLM: MAE = 1.84 weeks, *r* = 0.75)

We found that the GCN fed with the true brain mesh achieved an accuracy of prediction with a mean absolute error (MAE) of 0.963 weeks (5.3% of the total age range) and a correlation coefficient r of 0.94, which were smaller than the errors of the other two classic predictive models (random forest [RF] and general linear model [GLM]). Our model’s accuracy was also better than the CNN-based model (MAE = 1.05 weeks, *r* = 0.89) and the GCN on the random connections (MAE = 1.1 weeks, *r* = 0.89, Fig. 3).

### Clinical implications of the brain age index

**Fig. 4:**
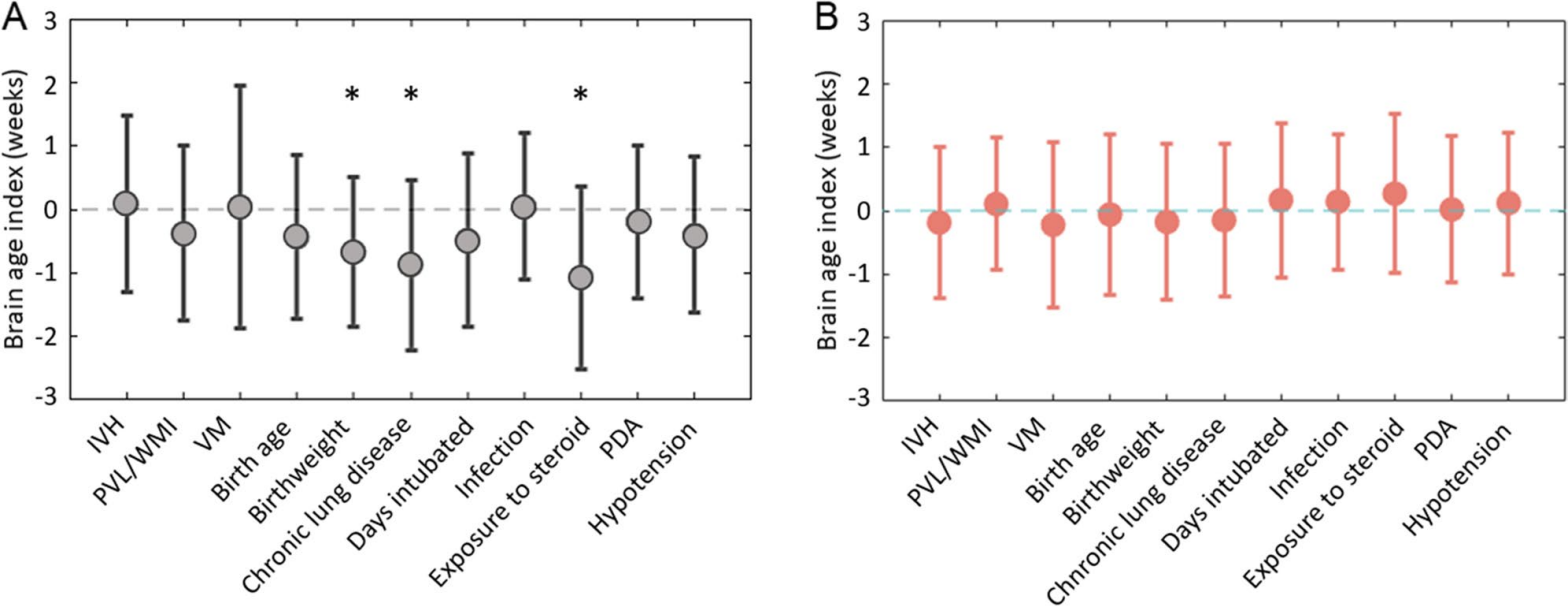
Lower BAI, representing impaired brain development, was associated with clinical factors in GCN-based brain age prediction model (**A**, left) rather than CNN-based model (**B**, right)

Univariate analyses showed that various clinical variables were associated with lower brain age index (BAI; t > 3.1; *p* < 0.05, false discovery rate [FDR] corrected; Fig. 4A). Postnatal steroid exposure (*p* = 0.007, FDR corrected) presented the strongest association with lower BAI. Chronic lung disease (CLD; *p* = 0.031, FDR corrected) and birthweight lower than 1000 g (*p* = 0.021, FDR corrected) were also significantly associated with lower BAI. No significant association was found between the BAI estimated from the CNN model and any clinical variables (Fig. 4B).

**Fig. 5:**
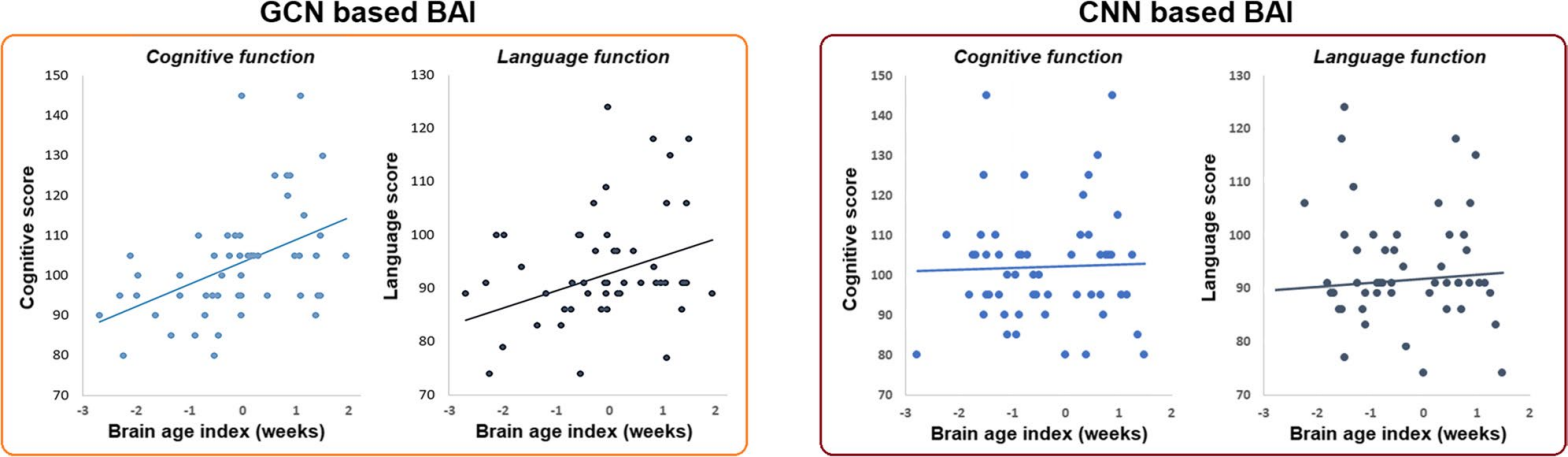
Correlation between BAI and neurodevelopmental outcomes (cognitive and language scores) at 30 months (left: measured using GCN; right: measured using CNN)

Correlation of BAI with cognitive, language, or neuromotor scores in Bayley-III Scales (Fig. 5 left) showed that a lower BAI at neonatal scan was significantly associated with lower cognitive performance (*r* = 0.41; *p* = 0.0025, FDR corrected) and lower language performance (*r* = 0.27; *p* = 0.042, FDR corrected). No significant associations were found between the BAI estimated from the CNN model and developmental outcomes at 30 months (Fig. 5 right).

**Fig. 6:**
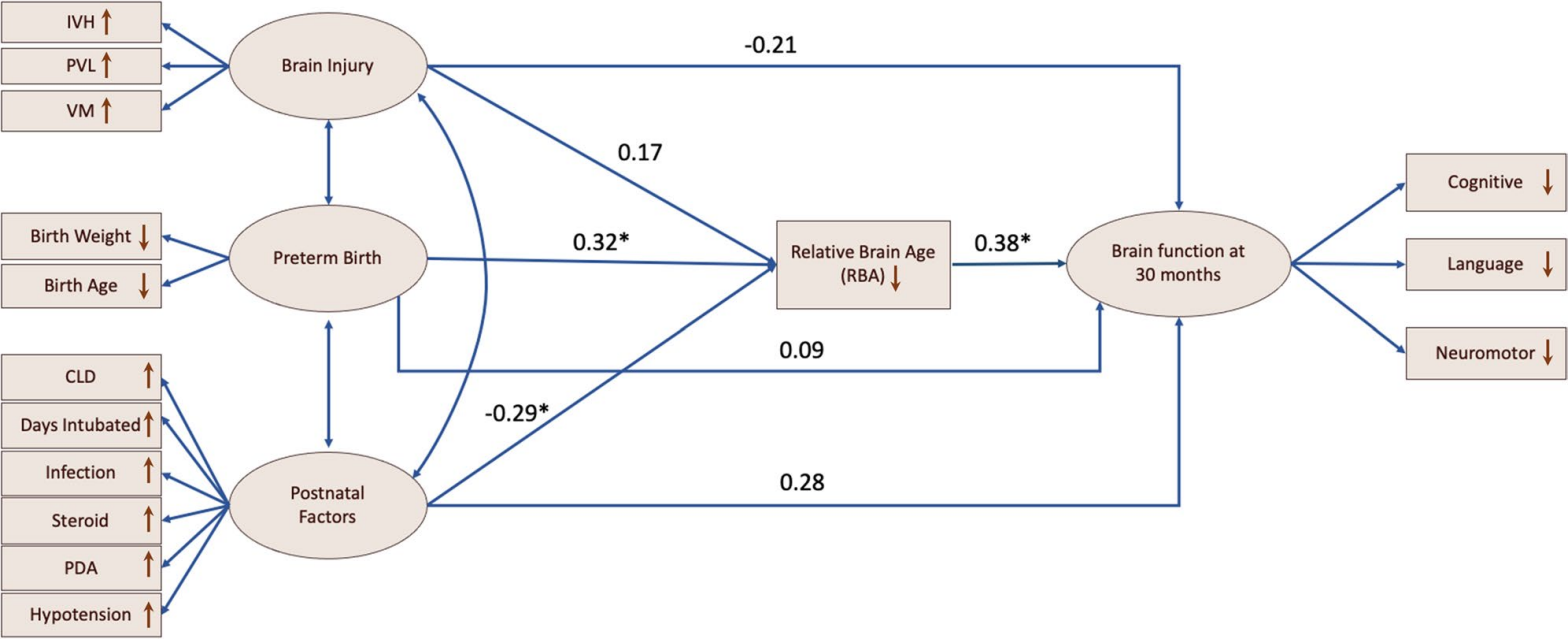
Results of path analysis. Rectangles represent manifest variables, and ellipses represent latent variables. Each single-headed arrow denotes a hypothesized unidirectional effect of one variable on another. Single-headed arrows represent the impact of one variable on another, and double-headed arrows represent covariances between pairs of variables. Numbers associated with effects are standardized regression coefficients. Asterisks refer to the paths that are statistically significant

The outlined SEM is shown in Fig. 6 (with standardized estimates). The *χ*^2^ model-fit statistics indicated a significantly acceptable model fit (*p* < 0.001). BAI mediated the pathway from preterm birth to brain functional development at 30 months. BAI also mediated the pathway from postnatal factors to brain functional development at 30 months (*p* < 0.05). The relationship between brain injury and brain development at 30 months was not significant, for either the direct pathway or the pathway mediated by BAI (*p* < 0.05).

## Discussion

### Clinical implications and applications

This study is the first to use structural equation modelling (SEM) to provide evidence of the temporal relationship among three stages of neurodevelopment: the context of preterm birth, postnatal brain development, and neurodevelopmental outcomes. That is, BAI at scan mediated the pathway from preterm birth and postnatal clinical factors, including birthweight, CLD, and exposure of postnatal steroids, to neurodevelopmental outcome at 30 months (Fig. 5). This suggests that brain morphological growth affected by preterm birth and postnatal factors is key to understanding later neurodevelopmental impairment. SEM did not provide sufficient evidence of a significant relationship between perinatal brain injuries and neurodevelopment outcomes. The underlying pathophysiology of how these clinical factors impair neurodevelopment may be attributed to both exogenous and endogenous factors that lead to brain insult from respiratory or circulatory insufficiency, hemorrhagic events, hypoxic-ischemic events, and inflammation [33–37].

Our results demonstrated that CLD has a strong association with impaired BAI. CLD primarily affects preterm infants who are exposed to prolonged mechanical ventilation and oxygen therapy for pulmonary complications [38, 39]. Chronic exposure to ventilation can result in oxygen toxicity, pulmonary inflammation, and lack of perfusion and oxygenation to the brain. Consequently, the immature brain becomes susceptible to hypoxia–ischemia, inflammation, germinal matrix injury, diffuse white matter injury, and diffuse gray matter injury [40, 41]. These complications, particularly diffuse white and gray matter lesions, contribute to neurodevelopmental impairment [42], consistent with the poor neurodevelopmental outcomes of CLD infants, including motor [43], language [44], and cognitive deficits [45–47].

Furthermore, our results revealed that postnatal exposure to steroids is associated with BAI impairment. Postnatal steroid therapy is traditionally provided to preterm neonates with BPD to reduce lung inflammation [48]. In our previous study, postnatal exposure to clinically routine doses of hydrocortisone or dexamethasone was associated with impaired cerebellar but not cerebral volumetric growth [49]. Using BAI based on cortical morphometrics and DL algorithms, however, we revealed additional adverse effects of postnatal glucocorticoids on cerebral growth. These results are consistent with previous findings demonstrating poor neurodevelopment following postnatal steroid exposure [50–52].

### Brain age index as a potential predictor of cognitive and language development in preterm children

The clinical utility of BAIs is further supported by their relationships with neurodevelopmental outcomes at 30 months (Figure S8). BAI metric was not associated with neuromotor scores at 30 months. A possible explanation is that the morphology in the motor cortex, particularly cortical folding, was already relatively mature in our cohort, given folding forms earlier than the late 3^rd^ trimester of gestation. Therefore, PBA based on cortical morphology extracted from these postnatal scans may not be sensitive to neuromotor impairment. Rather, previous studies showed that neuromotor impairment is associated with perinatal white matter brain injury [53, 54] and postnatal cerebellar growth [55].

### Brain age prediction using GCN

By capturing age-related morphological alterations throughout the third trimester, we proposed that GCN-based deep learning (DL) with surface morphological features can better predict individual brain age and neurodevelopmental outcomes. To date, PBA was derived from machine learning methods fed with volumetric images directly [14] or structural connectome metrics [16]. However, image-fed CNNs may not be capable of detecting the morphology that varies along the cortical manifold, which appears to be a sensitive gauge of early neurodevelopment.

To address these limitations, we incorporated morphological features extracted from the cortical surface and surface topology into our GCN and found that its prediction accuracy is superior to state-of-the-art methods, including an image-fed CNN model. Our GCN model revealed the underlying association of brain maturation with clinical variables and developmental outcomes, whereas the CNN model did not, likely indicating that the CNN requires more samples for training to avoid overfitting and enhance prediction.

### Limitations, future directions, and conclusions

Alterations in PBA could be a surrogate for brain developmental status. Notably, the brain age index is frequently influenced by various factors, giving rise to significant false positives and false negatives when assessing associations between BAI and other measures [56]. In this study, we only corrected the linear bias from the brain age index. As suggested by Smith et al [32], it is necessary to not only remove the linear dependency of brain age index on age but also the nonlinear dependence, especially the quadratic dependency of brain aging (as a function of age).

The data set used in this study was large but composed of heterogenous groups from two data sets, and the data from UCSF was acquired with different protocol/strength MRI. Although we performed harmonization, the number of population performed clinical evaluation and follow-up neurodevelopmental assessment were relatively small.

Further external validation will be performed in future studies. There is a huge time gap between the neonatal period and infancy at 30 months, which may limit the prediction ability and should be evaluated in a large sample study in the future.

It is important to approach SEM with a degree of caution, particularly when used for exploratory analyses. SEM is best employed when there are well-defined hypotheses about the relationships among variables. One of the limitations of SEM is that it assumes linearity and normality in the relationships among variables. It also requires a large sample size to ensure the stability and validity of its results. These are both marginally satisfied in this study. Furthermore, SEM’s capacity to provide plausible models does not prove that these models reflect true underlying processes. In light of these considerations, it would be prudent to moderate the claims made about SEM in this study.

Additional limitations in testing DL models in this study include the generalizability, and the interpretability of the model (including the use of uncertainty or confidence metrics) used in this study was not explicitly discussed, which will be guided by a checklist such as MAIC-10 in the future.

Despite these limitations, our study proposes a novel GCN that uses morphological features and topological patterns to predict brain age. The PBA, in turn, better explained neonatal developmental trajectory by linking pre-scan clinical factors and post-scan neurodevelopmental outcomes. Altogether, these findings provide the basis for future investigations aiming to extend the PBA measurement to practical clinical applications, such as the individualized prediction of neurodevelopmental outcomes.

### Supplementary information

Below is the link to the electronic supplementary material.

Supplementary file1 (PDF 1261 KB)

## Abbreviations

BAI: Brain age index
CLD: Chronic lung disease of prematurity
CNN: Convolutional neural network
dHCP: The developing Human Connectome Project
DL: Deep learning
FDR: False discovery rate
GA: Gestational age
GCN: Graph convolutional network
GLM: General linear model
GM: Gray matter
IVH: Intraventricular hemorrhage
MAE: Mean absolute error
NEC: Necrotizing enterocolitis
PBA: Predicted brain age
PDA: Patent ductus arteriosus
PMA: Postmenstrual age
PVL: Periventricular leukomalacia
RF: Random forest
ROI: Region of interest
SEM: Structural equation models
VM: Ventriculomegaly
WM: White matter

## Declarations

### Guarantor

The scientific guarantor of this publication is Hosung Kim.

### Statistics and biometry

One of the authors, Mengting Liu, has significant statistical expertise.

### Study subjects or cohorts overlap

Some study subjects or cohorts have been previously reported in the following:

Paper 1: “Hippocampal asymmetry of regional development and structural covariance in preterm neonates,” and Paper 2: “Disruption and compensation of sulcation-based covariance networks in neonatal brain growth after perinatal injury.”

The topic of Paper 1 is how preterm birth may exert an impact on hippocampal development in neonates, while Paper 2 is about how brain injury in preterm birth may alter the topological organization of brain structural network in neonates. Our current study, however, focuses on brain age prediction in neonates, which is a completely different research topic compared to the previously published work.

The manuscript has a pre-print https://doi.org/10.1101/2021.05.15.444320

### Methodology

• retrospective

• cross-sectional study

• multicenter study
