# Supplementary material for: Brain age predicted using graph convolutional neural network explains neurodevelopmental trajectory in preterm neonates
Source: Eur Radiol. 2023 Nov 14;34(6):3601–11. doi: 10.1007/s00330-023-10414-8 (PMC11166741; doi:10.1007/s00330-023-10414-8)
Supplement: Supplementary file 1 — Supplementary file1 (PDF 1261 KB) [file 330_2023_10414_MOESM1_ESM.pdf]

Brain age predicted using graph convolutional neural network explains neurodevelopmental trajectory in preterm neonates

Electronic Supplementary Material

Methods S1: Brain Injury

The severity scores were generated for IVH using the scoring system of Papile (0: absent; 1: germinal matrix hemorrhage; 2: IVH; 3: IVH with hydrocephalus; 4: parenchymal hemorrhage, usually periventricular hemorrhagic infarction)[1] and WMI (0: absent; 1: 2 mm; 3: >5% hemisphere) using established criteria.[1, 2] Subsequently, IVH scores were binarized with “mild” representing grades 1– 2, and “severe” representing grades 3–4; WMI and VM were categorized as “mild” for grade 1 and “severe” for grades 2– 3. For subjects with multiple MR examinations, the highest (most severe) score in each category was used for analysis. In the current study, we merged infants with mild injuries and those with no injury into one none-mild injury group since the two groups exhibited no significant differences in the following analyses.

Table S1. Definition and categorization of clinical variables for statistical analysis.

Factors	Definition	Grouping	
IVH	Papilie et al. 1978	grade 0-2 (143)	3-4 (25)
VM	Miller et al. 2003	grade 0-1 (114)	2-3 (18)
PVL (WMI)	Miller et al. 2003	grade 0-1 (152)	2-3 (56)
Birth_Weight	gram	>=1000 (72)	1-999 (93)
Birth_Age	weeks	>=28 (83)	<28 (87)
Days_Intubated	Mechanical ventilation and intubation	0-5 (87)	>=5 (57)
PDA	Patent ductus asteriosus	No (69)	Yes (87)
CLD		No (107)	Yes (74)
Neonatal_Infection	Culture + or – sepsis, and/or meningtis	No (81)	Yes (89)
Postnatal_Steroid	Exposure to postnatal hydrocortisol	No (134)	Yes (21)
Hypotension	Requiring treatment with volume resuscitation and/ or vesopressors required	No (50)	Yes (103)

Table S2. Definition and categorization of clinical variables in dHCP dataset for statistical analysis.

Factors	Definition	Grouping	
IVH	Papilie et al. 1978	grade 0-2 (394)	3-4 (13)
VM	Miller et al. 2003	grade 0-1 (407)	2-3 (0)
PVL (WMI)	Miller et al. 2003	grade 0-1 (391)	2-3 (16)

Methods S2: Steps for NEOCIVET pipeline

The cortical surfaces were constructed using the NEOCIVET pipeline.[3-5] The pipeline began with general MR image pre-processing, including denoising and intensity nonuniformity correction. Then, the brain is extracted using a patch-based brain extraction algorithm (BEaST)[6] and registered to the MNI-NIH neonatal brain template (http://www.bic.mni.mcgill.ca/ServicesAtlases/NIHPD-obj2). Different types of brain tissue (GM, WM, and CSF) were thereafter segmented by a label fusion based on a joint probability between selected templates.[7] Next, the corpus callosum was segmented on the midline-plane and used to divide the WM into hemispheres. A marching cube-based framework was adopted to generate a triangulated mesh WM surface attached to the boundary between the GM and WM. After resampling to a fixed number of 81,920 surface meshes using the icosahedron spherical fitting, this surface was further fitted to the sharp edge of the GM-WM interface based on the image intensity gradient information. This allowed for the deformation while preserving the spherical topology of the cortical mantle. A CSF skeleton was then generated from the union of WM and CSFs. Pial surface was constructed by expanding the WM surface towards the skeleton as an intermediate pial surface. The intermediate pial surface further underwent a fine deformation to identify actual edges of sulcal CSF volumes using an intensity gradient feature model. Finally, the cortical thickness was estimated based on the Euclidean distance between the white matter and pial surface.

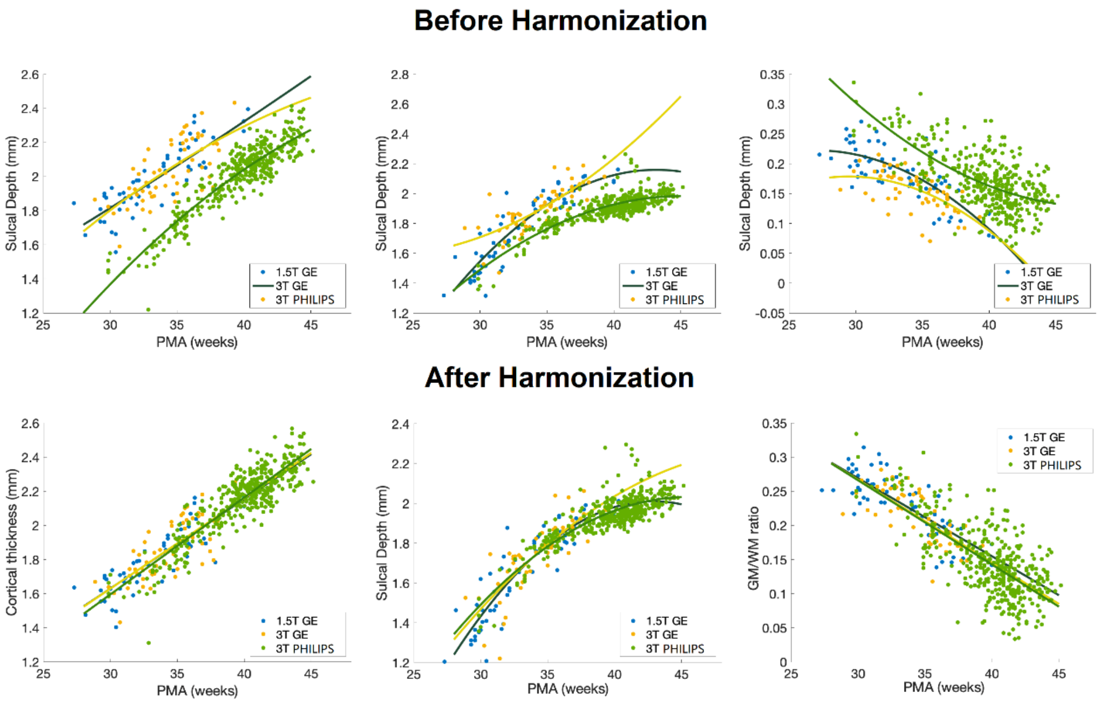

Figure S1. Comparison of three cortical features used in this study after the ComBat harmonization method. We applied ComBat to the 3 groups of scanner types - 1.5T GE (with 1x1mm voxel size), 3T GE (0.7x0.7mm), and 3T Siemens (0.6x0.6mm). Results demonstrate that ComBat can remove possible bias and generate a consistent trajectory of maturation from 3 different data sources as seen in the 3 different imaging features used for brain age prediction.

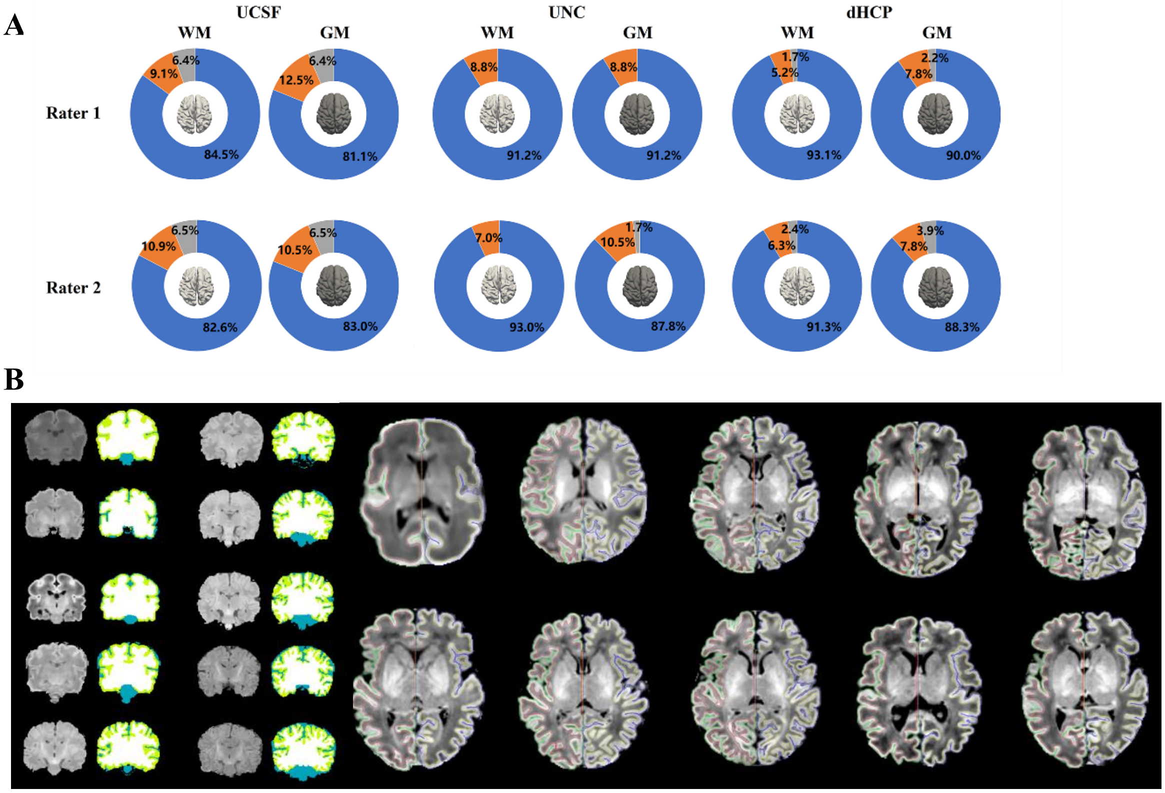

Figure S2. A) The newest NEOCIVET version was used in this study.[8] NEOCIVET was developed based on 3 datasets from UCSF (1.5T & 3T), dHCP (3T), and University of North Carolina (UNC, 3T). All key steps in the pipeline, including 3D U-Net models for brain and tissue segmentation, were trained using the 3 datasets. The surface reconstruction quality has been validated on these datasets by an expert neuroscientist whose scoring is provided in the figure (blue: good quality, orange: fair quality, gray: poor quality). B) Illustration of the output from the NEOCIVET pipeline. Left panel represents the tissue segmentation. The right panel displays the surface reconstruction: 10 individual surfaces are overlaid on MRI, which were reconstructed from the dHCP dataset.

Methods S3: Graph convolutional neural network (GCN) based brain age prediction

The proposed PBA model using GCN is illustrated in Figure 1. GCNs[9] are designed to exploit the underlying graph structure of the data. To this end, GCNs consider spectral convolutions on graphs defined as the multiplication of a signal with a filter in the Fourier domain.[10] The signal h on the graph nodes is filtered by g as:

$$g * h = \boldsymbol{U}(\boldsymbol{U}^T g \odot \boldsymbol{U}^T h) \quad (1)$$

where U is the Fourier basis of the graph Laplacian L, given by the eigen decomposition of L i.e., $\mathbf{L} = \boldsymbol{U\Lambda U}^T$, $\boldsymbol{\Lambda}$ is the ordered real nonnegative eigenvalue values vector of graph Fourier transform, $*$ is the convolution operator and $\odot$ denotes element-wise multiplication. The graph Laplacian L is defined as L: = D – W where the degree matrix D is a diagonal matrix whose ith diagonal element d_i is equal to the sum of the weights of all the edges connected to vertex i as $D_{ii} = \sum_j W_{ij}$. W is a binarized adjacency matrix encoding the connection between vertices, where 1 represents a connection between two vertices in brain mesh and 0 otherwise. After normalization, the graph Laplacian is defined as $\mathbf{L} = I_n - \mathbf{D}^{-1/2}\mathbf{W}\mathbf{D}^{-1/2}$ where I_n is the identity matrix.

Vertices on graph are re-arranged such that a graph pooling operation becomes as efficient as 1D pooling. Fake nodes, or disconnected nodes, are added to construct a balanced binary tree from the coarsest to finest level to make the pooling operation very efficient without losing information.

Mean squared error (MSE) was used as the loss function with an Adam optimizer, the empirically determined set of parameters with a learning rate of 10^{-6}, an L2 regularization parameter of 10^{-8}, and a batch size of 2 were applied.

Methods S4: number of nodes in GCN

We down-sampled 81,924 vertices on cortical surfaces using the icosahedron downsampling to investigate the accuracy of the GCN-based brain age prediction while saving computational time in the training of GCN. We thus feed each of the brain meshes that were down-sampled with 324, 1,284, 5k and 20k vertices to the GCN model respectively. The number of 1,284 was chosen to use in the following analysis by a compromise of the computational accuracy and computational time (Figure S3).

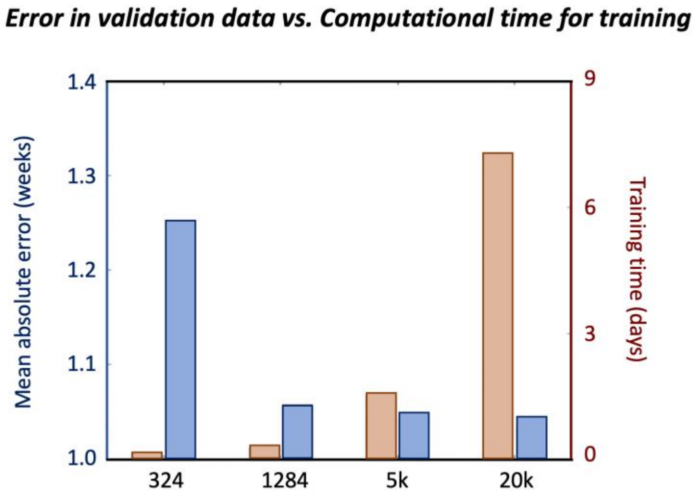

Figure S3. The number of 1,284 was chosen to use in the following analysis by a compromise of the computational accuracy and computational time

Methods S5: Convolutional neural network (CNN) based brain age prediction

To compare our cortical surface-based GCN model with conventional image-based deep learning model, we also built a brain age prediction model by applying a 2D aggregated CNN-based deep learning model (Gupta et al., ISBI 2021) to T1 MR images directly. This model was fed a 3D scan as input and encoded each image slice using a 2D-CNN encoder. Next, it combines the slice encodings using an aggregation module, resulting in a single embedding for the scan. Finally, this embedding was passed through the feed-forward layers to predict the brain age. The model was trained end-to-end using MSE loss. More details of the used network architecture can be found in (Gupta et al., 2021).[11] This model has been proved to be better than conventional 3D-CNN based model in adults brain age prediction study (Gupta et al., 2021). This model has a shallow network architecture and might be better for learning brain age from a small sample dataset. A recent paper from Smith's group also concluded that a shallow neural network performs more favorably for the brain age prediction task (Peng et al., Medical Image Analysis 2021).[12]

Methods S6: Hyperparameter tunning of all the brain age prediction models used in this study.

For the CNN model, we first used the hyperparameters recommended by the author in their original paper (Gupta et al., 2021)[11] and then varied these parameters from this initial setting and tested them in separate runs, which allowed us to achieve the best accuracy of the CNN model.

For the Random Forest model, the computational time is quite short. Hence, to tune the parameters, we tested various combinations of parameters, including the number of trees, maximum levels in each tree, maximum number of features used for splitting a node, and the need for bootstrap analysis. Our reported results are derived from the combination of parameters that led to the highest PBA accuracy.

The optimal setting of hyperparameters for the GCN model has not yet been documented in previous studies. Thus, we tried to achieve the best accuracy by testing various parameters, including the number of layers, the kernel size, the number of filters, pooling approaches, etc. Due to the huge computational burden in deep learning algorithms, however, we did not focus on finding the best combination of parameters. Instead, we independently varied and tested each parameter while fixing others. As a result, we found that the GCN model obtained the best performance when it had 3 convolutional and pooling layers, with the kernel size of 5 (vs. 3 or 10), the number of filters to be [8, 16, 32] (vs. 16, 32, 64), the pooling size of 2 and the max-pooling approach (vs. average pooling). We also found that the outputs based on different settings were only slightly different. Notably, a larger variation in output performance was observed when using different random initialization and nested 5 folds cross-validation models. We thus built several models (100 initialization X 5 folds) and averaged them.

Methods S7: Carefully designed cross-validation strategy.

One-fifth of the sample was excluded from the training step and reserved as unseen testing data (Figure S4-left). The other four fifths were entered into the nested 5-folds cross-validation loop as training and validation samples presented in figure S4-left or the figure below for your convenience. The nested cross-validation set underwent separate training-validation processes using its own 5 folds to avoid overfitting (Figure S4-middle). For each fold of the processes, four-fifths of the sample was grouped as training samples, and the remaining one-fifth was used as validation samples. For each fold, the trained model was applied to the unseen test data to generate predicted ages. Also, we ran the cross-validation model 100 times using the random initialization step in the GCN to remove potential bias due to different initial parameter settings. The final predicted ages were calculated by averaging the output from the 5x100=500 models.

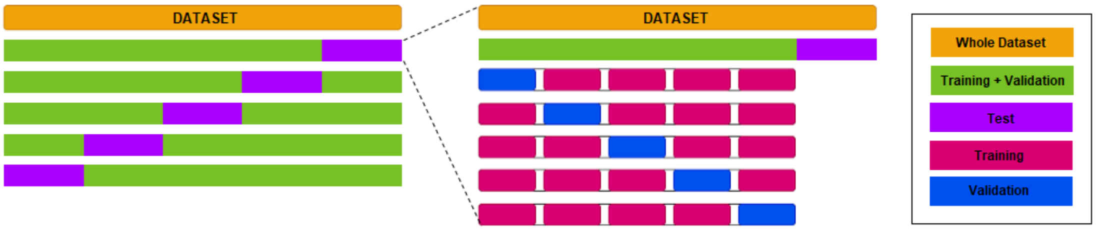

Figure S4. The training strategy of GCN in our study.

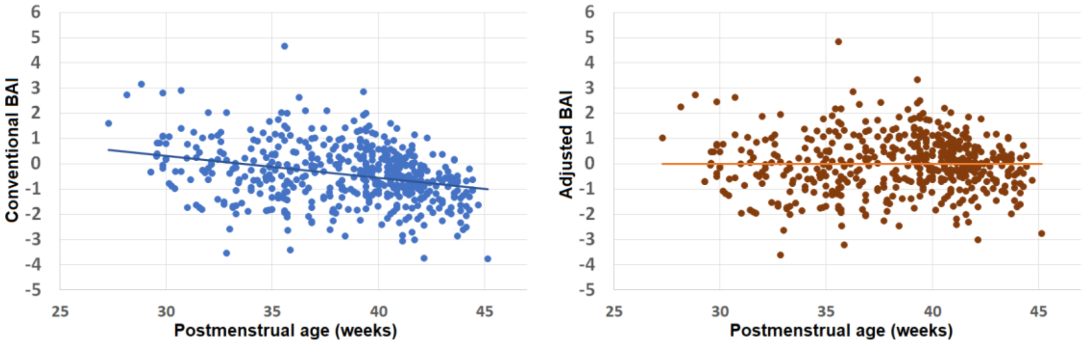

Figure S5. The conventional RBA is negatively associated with true age (left, $p < 0.05$), while this effect is corrected in new RBA (right).

Table S3. The performance of the age prediction (errors in weeks for MSE, MAE and SDAE).

	MSE	MAE	SDAE	r-value
Cortical thickness	2.501	1.891	1.682	0.65
Sulcal depth	2.477	1.882	1.675	0.66
GM/WM intensity ratio	2.889	2.272	1.775	0.44
General linear model	2.432	1.838	1.655	0.75
Random forest regression	1.796	1.273	1.165	0.85
GCN with random mesh	1.593	1.071	1.027	0.89
CNN	1.578	1.056	1.011	0.89
GCN with true mesh	**1.463**	**0.963**	**0.902**	**0.94**

Abbreviation: MSE = mean squared error; MAE = mean absolute error; SDAE= absolute standard deviation

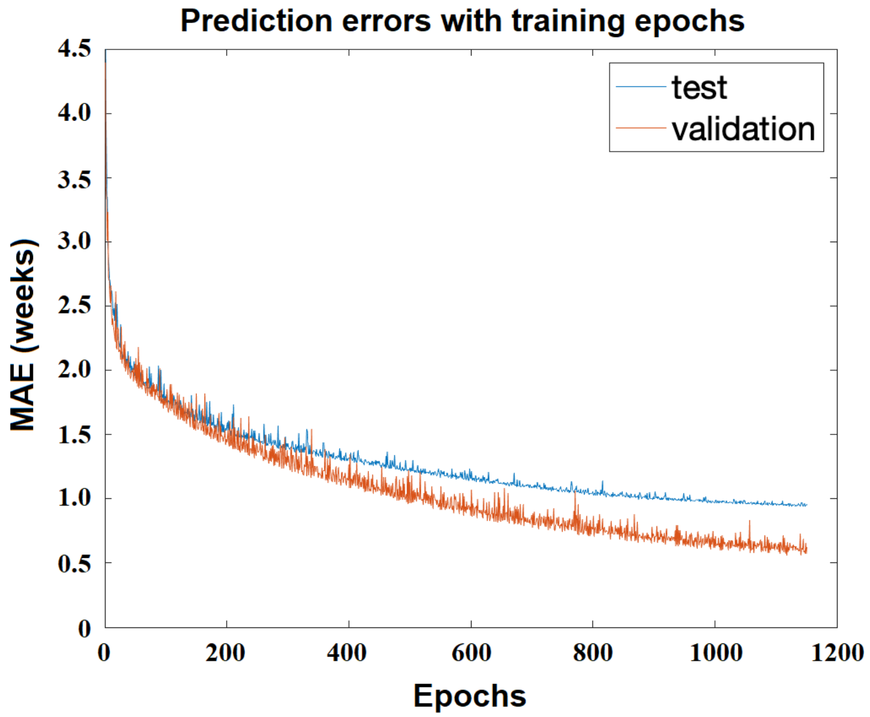

Figure S6. The epochs converging along with the iteration for our training and validation datasets.

Methods S8: Association of clinical variables with the morphological and imaging features used as the input to brain age prediction models, such as cortical thickness, sulcal depth, and GM/WM intensity ratio.

To compare whether the brain age index, which is based on three cortical thickness, sulcal depth, and GM/WM ratio, is more sensitive to the brain abnormalities than the three features respectively, we assessed the association between three cortical features and clinical factors. Specifically, we first averaged each of the three cortical features across all the vertices per individual. Similar to brain age index, we then converted each feature into a global normative index to indicate how the cortical features may deviate from the normal developmental trajectories. To do this, we ran a normative modelling using Gaussian process regression with a 5-fold cross validation to predict the cortical features while correcting for the effects of age at scan and sex. The predicted values are in a z-score quantifying abnormality deviating from the normal developmental trajectory. Then, a similar analysis, as shown in Figure 3, was conducted on z-scores which were calculated from each cortical feature. The brain age index and the cortical features exhibited similar

patterns in relation to various clinical variables to an extent. However, the brain age index had the best sensitivity compared to single cortical features as it showed the greatest number of significant associations.

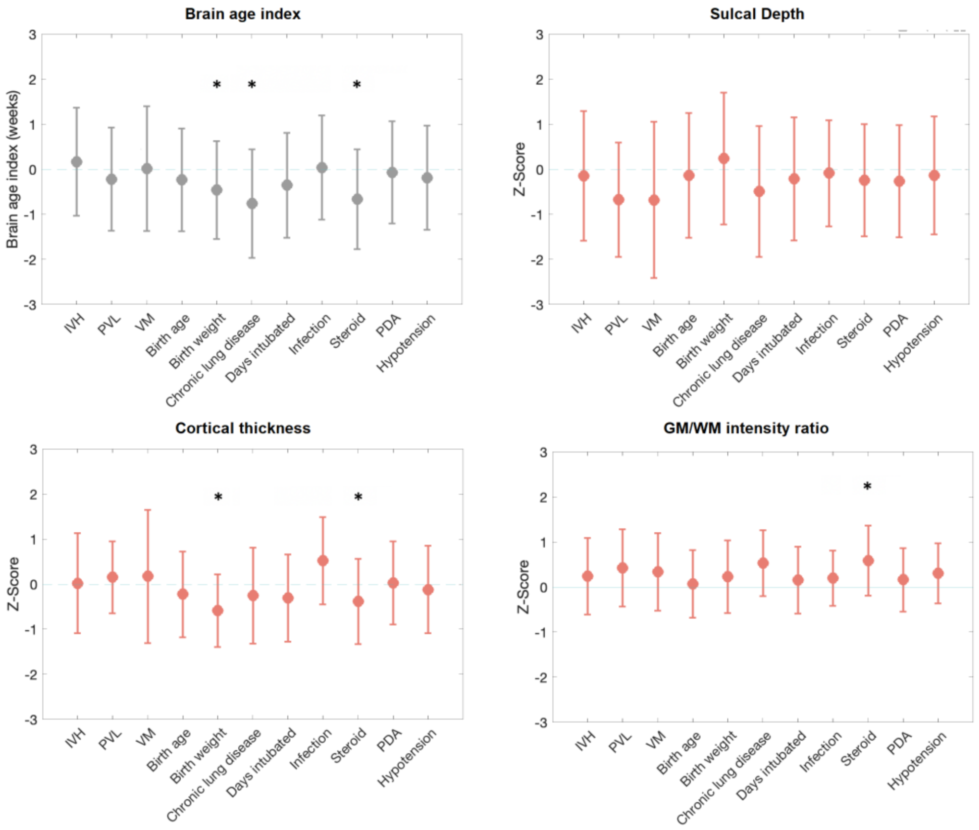

Figure S7. Mean absolute error map for regional BAIs predicted using GCN models.

Methods S9: Structural Equation Modelling

We built structural equation models (SEM) that impute relationships between latent variables. Based on the hypothesized latent risk variables and timeline in Figure 2, we analyzed multiple relationships/paths between severity of preterm birth, perinatal injuries, pre-scan post-natal factors, BAI at postnatal scan and neurodevelopmental outcome scores at 30 months.

This analysis, which was designed to identify the clinical variables and their paths leading to adverse neurodevelopmental outcomes, was conducted only on 50 MRI images from UCSF dataset including baseline and follow-up scans at 30 months from 38 preterm survivors.

By constructing the SEM, we tested our hypothesized model (Figure 2) using the previously mentioned data and determined the strength and significance of each hypothesized path. We estimated all parameters using weighted least squares with standard errors and mean- and variance-adjusted test statistics with a full weight matrix (WLSMV), which yields parameter estimates and standard errors that are robust to violations of multivariate normality 34. We used the $\chi 2$ statistic fit indices to evaluate whether the model fit to data well. We reported standardized parameter estimates computed using the WLSMV estimation to enable more direct comparisons of the effects for different pathways to neurodevelopmental outcomes.

To begin, we used the confirmatory factor analysis (CFA) to model latent variables summarizing a subset of pathologies. As a priori, we hypothesized a latent variable for 1) preterm birth measures, informed by birth age and birthweight; 2) the presence of perinatal injuries; 3) postnatal conditions/treatments, informed by exposure to steroids, hypotension, infection, PDA, days intubated, and CLD; 4) neurodevelopmental outcome at 30 months (cognitive, language, and motor scores).

Results S1: Accuracy of regional brain age prediction

Based on 5-fold cross-validation, age prediction for regional meshes resulted in a range of prediction accuracy with MAE of 1.19-2.42 weeks (Figure S10). We also found that the 9 smallest ROIs that contained less than 500 vertices (left, right olfactory cortices; left and right temporal poles of middle temporal gyrus; left and right Heschl gyri; left and right middle frontal gyri – orbital portion, left and right posterior cingulate cortex) displayed much larger MAEs (>1.75 weeks), possibly explaining the lack of fitting in the model due to a small number of their feature samples. Other 65 ROIs (>500 vertices) showed more reliable performance with MAEs less than 1.5 weeks. The pattern of prediction accuracy for cortical regions was hemispherically symmetric. The right post central cortex showed the most accurate prediction for NMI brains (MAE: 1.1 weeks), followed by the left superior frontal cortex and left superior temporal cortex (1.21 weeks), which were slightly larger than the prediction error computed using the whole brain surface data (1.06 weeks). These three regions also presented the highest slopes of brain age over the chronological age, which indicated their faster development.

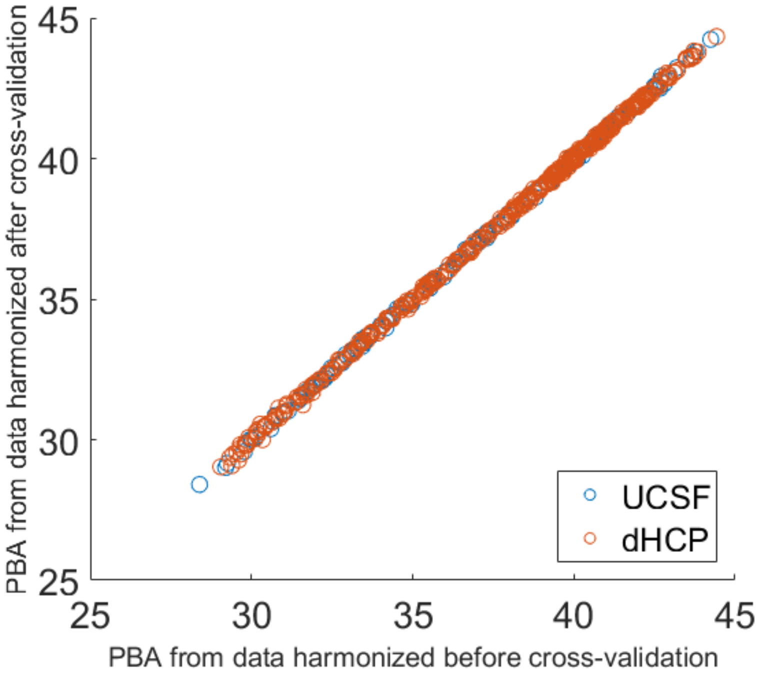

Figure S8. Predicted brain age using cortical feature harmonized by combat before and after splitting them into training and test. Notably, the MAE reported here is slightly different from that in manuscript because the MAE value in the manuscript is based on the average of 100 outputs (more details can be found in our response to your next question). Given the very small differences between the two models, we decided to keep the original results, as changing the model would be extremely time-consuming and yet not change the main message of the paper.

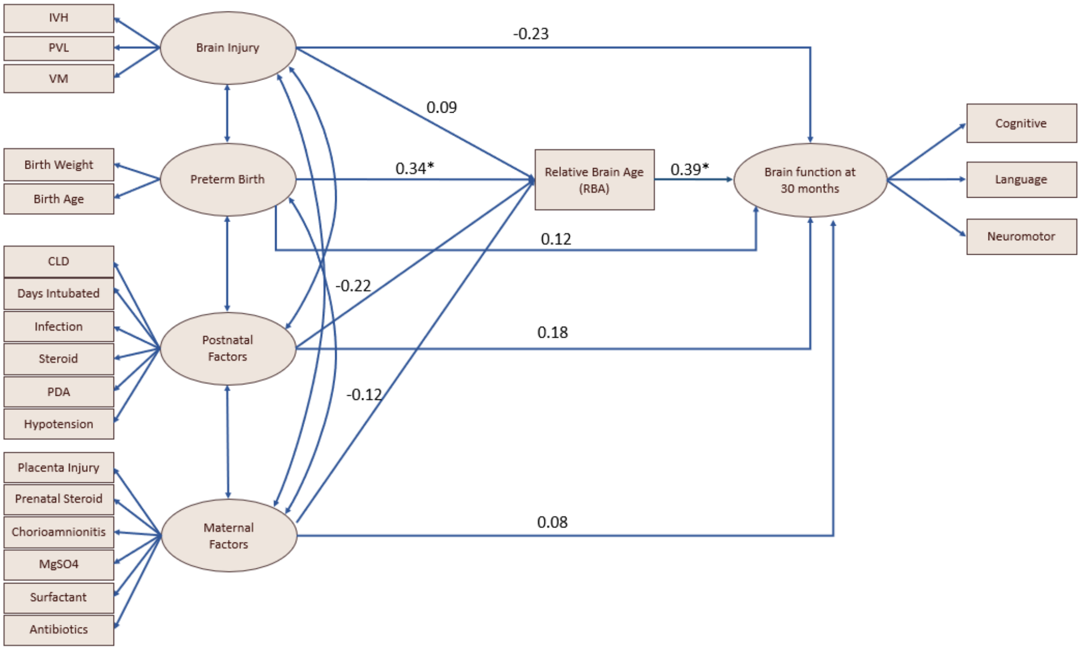

Figure S9. Results of path analysis including the maternal risk factors. Rectangles represent manifest variables, and ellipses represent latent variables. Each single-headed arrow denotes a hypothesized unidirectional effect of one variable on another. Single-headed arrows represent the impact of one variable on another, and double-headed arrows represent covariances between pairs of variables. Numbers associated with effects are standardized regression coefficients. Asterisks refer to the paths that are statistically significant.

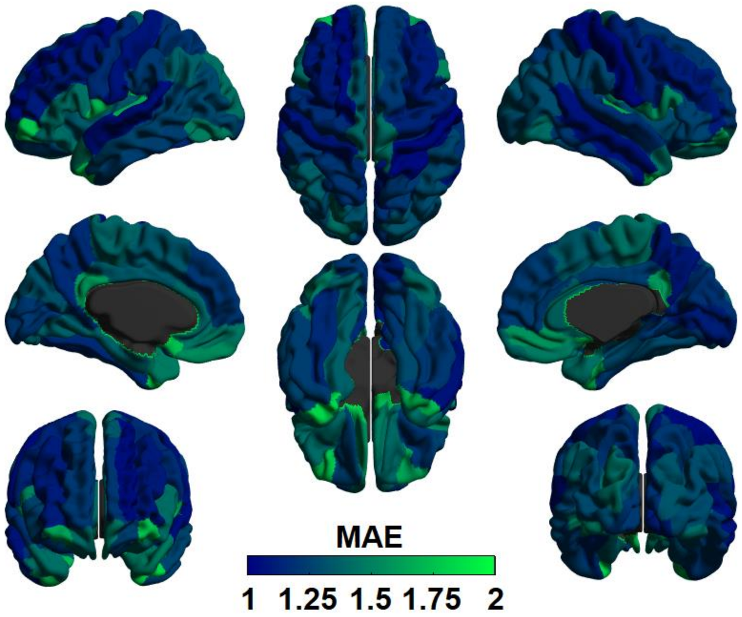

Figure S10. Mean absolute error map for regional BAIs predicted using GCN models.

References:

1. Papile L-A, Burstein J, Burstein R, Koffler H. Incidence and evolution of subependymal and intraventricular hemorrhage: a study of infants with birth weights less than 1,500 gm. *Journal of pediatrics* 1978; **92**(4)**:** 529-534.

2. Miller SP, Cozzio CC, Goldstein RB, Ferriero DM, Partridge JC, Vigneron DB *et al.* Comparing the diagnosis of white matter injury in premature newborns with serial MR imaging and transfontanel ultrasonography findings. *American Journal of Neuroradiology* 2003; **24**(8)**:** 1661-1669.

3. Kim H, Lepage C, Maheshwary R, Jeon S, Evans AC, Hess CP *et al.* NEOCIVET: Towards accurate morphometry of neonatal gyrification and clinical applications in preterm newborns. *Neuroimage* 2016; **138:** 28-42.

4. A Skeleton and Deformation Based Model for Neonatal Pial Surface Reconstruction in Preterm Newborns. *Proceedings of the 2019 IEEE 16th International Symposium on Biomedical Imaging (ISBI 2019)*2019. IEEE.

5. Liu MT, Lepage C, Kim SY, Jeon S, Kim SH, Simon JP *et al.* Robust Cortical Thickness Morphometry of Neonatal Brain and Systematic Evaluation Using Multi-Site MRI Datasets. *Front Neurosci-Switz* 2021; **15**.

6. Eskildsen SF, Coupe P, Fonov V, Manjon JV, Leung KK, Guizard N *et al.* BEaST: Brain extraction based on nonlocal segmentation technique. *Neuroimage* 2012; **59**(3)**:** 2362-2373.

7. Wang HZ, Suh JW, Das SR, Pluta JB, Craige C, Yushkevich PA. Multi-Atlas Segmentation with Joint Label Fusion. *Ieee T Pattern Anal* 2013; **35**(3)**:** 611-623.

8. Liu M, Lepage C, Kim SY, Jeon S, Kim SH, Simon JP *et al.* Robust cortical thickness morphometry of neonatal brain and systematic evaluation using multi-site MRI datasets. *Front Neurosci-Switz* 2021; **15:** 218.

9. Defferrard M, Bresson X, Vandergheynst P. Convolutional neural networks on graphs with fast localized spectral filtering. *Advances in neural information processing systems* 2016; **29:** 3844-3852.

10. Shuman DI, Narang SK, Frossard P, Ortega A, Vandergheynst P. The emerging field of signal processing on graphs: Extending high-dimensional data analysis to networks and other irregular domains. *IEEE signal processing magazine* 2013; **30**(3)**:** 83-98.

11. Improved Brain Age Estimation with Slice-based Set Networks. *Proceedings of the 2021 IEEE 18th International Symposium on Biomedical Imaging (ISBI)*2021. IEEE.

12. Peng H, Gong W, Beckmann CF, Vedaldi A, Smith SM. Accurate brain age prediction with lightweight deep neural networks. *Medical image analysis* 2021; **68:** 101871.
